# Role of meditation on the essence of self in the psychological profile, quality of life and lifestyle – a comparative study

**DOI:** 10.1016/j.clinsp.2025.100682

**Published:** 2025-06-02

**Authors:** Paula Ricci Arantes, Tatiana Caccese Perrotti, Susan Andrews, Ernesto Sasaki Imakuma, Rosana Aparecida de Oliveira Maurelli, Emmanuel A. Burdmann

**Affiliations:** a LIM 44, Departamento de Radiologia, Faculdade de Medicina, Universidade de São Paulo (FMUSP), São Paulo, SP, Brazil; b Centro Medicina Mente/Corpo, Departamento de Clínica Médica, Faculdade de Medicina, Universidade de São Paulo (FMUSP), São Paulo, SP, Brazil; c Instituto Visao Futuro, Porangaba, SP, Brazil; d LIM 12, Disciplina de Nefrologia, Faculdade de Medicina, Universidade de São Paulo (FMUSP), São Paulo, SP, Brazil

**Keywords:** Meditation, Stress, Resilience, Empathy, Quality of life, Lifestyle

## Abstract

**Objectives:**

Meditation has been shown to influence physical and psychological health and well-being, modulating stress response, resilience, emotional regulation, mood, empathy, compassion, and improving quality of life. The authors aim to characterize the psychosocial profile of experienced practitioners of Meditation on the Essence of Self, the Gurusakásha, whose technique is based on visualization and mantra from the Indian tantric tradition.

**Methods:**

Meditation experts (*n* = 23) were paired to an age-, gender- and educational level-matched control group (*n* = 29). Clinical data (presence of baseline diseases, use of medications, habits), stress level (PSS), resilience (CD-RISC), subjective mood states (VAMS), empathy (IRI), quality of life (WHOQOL-BREF), and general lifestyle profile (PEVI) were compared between groups.

**Results:**

Experienced meditators had lower levels of perceived stress, and better scores on resilience, empathic concern, quality of life, and lifestyle profile suggesting that Meditation on the Essence of Self improves health and well-being.

**Conclusions:**

These results suggest that experienced meditators on the Essence of Self have higher levels of psychological well-being and quality of life when compared to non-meditators.

## Highlights

• First research to investigate meditators’ profile for *Gurusakásha* meditation.
• Meditators had higher levels of psychological well-being and quality of life.
• Third phase meditation may impact stress, resilience, lifestyle, and quality of life.

## Introduction

Meditation is a theme of increasing interest in the medical field nowadays. Different types of meditation have been shown to play a role in physical and psychological health and well-being,^1^, ^2^, ^3^ improving quality of life,^1^^,^^2^ modulating stress response,^1^^,^^4^, ^5^, ^6^ resilience,^4^^,^^5^^,^^7^ emotions,^6^^,^^8^ mood,^1^^,^^3^^,^^6^ empathy and compassion.^5^^,^^9^

As previously reported^10^ there were distinct phases of meditation research, beginning with mindfulness/presence meditation, which have become the dominant paradigm for clinical research, passing through love and compassion meditation, and the more recent interest in ancient meditative practices like Tantra. The philosophy and practices of Tantra are traditions developed on the Indian subcontinent possibly 7.000 years ago, aspiring for mind expansion through practices focusing on the sublime.^11^ However, in the 20th century, a distorted tantric practice was introduced in the United States creating a false connection to sex. This Western tantra does not represent the original practice.

The present study focused on the technique *Gurusakásha* or meditation on the Essence of Self which is at the same time deep and simple.^11^ In Sanskrit, guru means “one who removes the darkness from the mind” and Gurusakásha means “near the Guru” or “under the Guru’s shelter”. In ancient Tantric tradition, the Guru is an expression of a higher consciousness, an internal, spiritual guide to dispel the darkness and negativity from the mind and realize one’s essential Self. It is categorized as a third-phase meditation,^10^ since it is based on a combination of visual imagery, mantra, and respiratory control. The authors hypothesize it has positive health implications, especially on resilience, stress management, quality of life, and lifestyle choices.

This is an exploratory study assessing and comparing the psychosocial profile of experienced Essence of Self meditators with a control group. The aim is to investigate if there are differences in the psychosocial profile between meditators and non-meditators, specifically in stress level, resilience, subjective mood states, quality of life, and general lifestyle profile, by validated instruments. This study conforms to the STROBE Statement.

## Materials and methods

### Ethics

This unicentric, observational, transversal, prospective study was approved by the Ethics Committee of the FMUSP (#4.163.528).

All participants signed an informed consent, and received no funding for the participation, except for a lunch box. The data was anonymized for processing.

### Participants

Meditation Experts (ME) were recruited from all over the country practitioners of the “Instituto Visão Futuro” (https://www.visaofuturo.org.br), a Faculdade de Medicina da Universidade de São Paulo (FMUSP) Mind-Body Center partner. They were selected as experts according to a meditation performance evaluation by their senior instructor.

The short Meditation on the Essence of Self includes an early morning practice, which can be repeated throughout the day. It consists of a visualization of a lotus white flower near the top of the head, in the infra bregmatic area inside the skull, with a visualization of your personal spiritual master, while mentalizing an internal mantra, and its meaning.^11^

Control participants (CO) were recruited via social media group advertisements in the local community. The advertisement specifically recruited participants without prior meditative experience. They were selected and paired to the ME in age, gender, and educational level.

All participants fit the inclusion criteria: age over 18 years old, voluntary engagement after invitation, agreement to not being funded, and for the ME group, have been practicing meditation for >30 hours per month. The exclusion criteria included current or previous neurological diseases, neurosurgery or head trauma, and obesity (BMI ≥ 35 kg/m^2^).

### Evaluated parameters

Age, gender, and educational data (number of years of total education) were accessed from the digital questionnaires and used for pairing the CO and ME groups.

The clinical forms contained information about the presence of hypertension, diabetes, thyroid disorders, smoking, alcoholism, other diseases, medicine intake, and physical exercise.

The stress level was evaluated by the validated Brazilian version of the Perceived Stress Scale (PSS),^12^ which has good construct validity and psychometric properties (α = 0.82).

Resilience was assessed by the validated Brazilian-Portuguese version of the Connor-Davidson Resilience Scale (CD-RISC).^13^ CD-RISC has very good internal consistency (α = 0.93) and intraclass correlation coefficient (ICC = 0.86).

Subjective mood states were assessed using the Visual Analogue Mood Scale (VAMS) translated into Brazilian Portuguese.^14^ The 16 items of the VAMS are grouped into four factors: anxiety, mental sedation, physical sedation, and other feelings and attitudes.

Empathy was evaluated using the validated Brazilian Portuguese version of the Interpersonal Reactivity Index of Davis (IRI)^15^ which has three subscales: emotional (empathic concern), cognitive (perspective taking), and behavioral (personal distress). IRI has adequate reliability (IRI α = 0.75).

Quality of life and its domains (physical and psychological health, social relationships, and environment) were assessed by the validated Brazilian Portuguese version of the World Health Organization Quality of Life-BREF questionnaire (WHOQOL-BREF).^16^ This questionnaire has good psychometric properties including internal consistency (α = 0.91) and test-retest reliability (0.69 to 0.81).

General lifestyle and five of its dimensions, nutrition, social relationships, stress management, physical activity, and preventive behavior, were evaluated by the Individual Lifestyle Profile (PEVI).^17^^,^^18^ General lifestyle profile scores are classified into five categories: excellent, good, regular, below the average, and bad; and dimension scores are classified into three categories: positive, regular, and negative. PEVI has reasonable internal consistency (α = 0.78) and construct validity.

### Statistical analysis

Statistical analysis was performed using Jasp version 0.11.1.

Data distribution was examined using the Shapiro-Wilk test. Continuous data are presented as the median (first and third quartiles) if they are not normally distributed or the mean ± standard deviation if they are normally distributed. Categorical data are shown as frequencies and percentages. Student’s *t*-test or the Mann-Whitney *U* test (MW) was used to compare continuous variables, as appropriate. MW was applied for categorical variables. Statistical significance was set at a 2-tailed p-value < 0.05.

The internal consistency reliability of each questionnaire was assessed by Cronbach’s alpha reliability coefficient and the measure was classified as the following: > 0.90 – Excellent, 0.80‒0.89 – Good, 0.70‒0.79 – Acceptable, 0.60‒0.69 – Questionable, 0.50‒0.59 – Poor, and < 0.5 – Unacceptable.^19^

## Results

The authors selected 23 ME. They had a mean of 15.1 years of meditation practice (SD = 14.9 years) and used to meditate 63.7 min per day (SD = 21.0 min). The CO was composed of 29 subjects.

All participants completed the totality of questionnaires.

Only physical activity practice was different between groups, performed by 96 % of the ME and 62 % of the CO (*p* = 0.004). All other clinical and sociodemographic characteristics were not significantly different between the two groups (Table 1).

**Table 1:** Socio-demographic and clinical characteristics of meditation experts and controls.

	Meditation experts n (%) or mean ± SD	Controls n (%) or mean ± SD	p
**Gender**			
Male	8 (34.8 %)	10 (34.5 %)	
Female	15 (65.2 %)	19 (65.5 %)	‒
**Age (years)**	46.3 ± 13.5	45.5 ± 14.7	‒
**Formal education (years)**	19.8 ± 4.3	18.2 ± 3.8	0.171
**Any physical activity practice**	22 (95.6 %)	18 (62.1 %)	0.004
**Medication intake**	7 (30.4 %)	10 (34.5 %)	0.757
**Comorbidities**			
Smoking	0 (0 %)	0 (0 %)	Variance=0
Alcoholism	0 (0 %)	1 (3.5 %)	0.369
Diabetes	0 (0 %)	0 (0 %)	Variance=0
Hypertension	0 (0 %)	3 (10.3 %)	0.112
Thyroid disorders	2 (8.7 %)	3 (10.34 %)	0.841
Other diseases	7 (30.4 %)	7 (24.1 %)	0.611

SD, Standard Deviation.

### Perceived stress

**Fig. 1:**
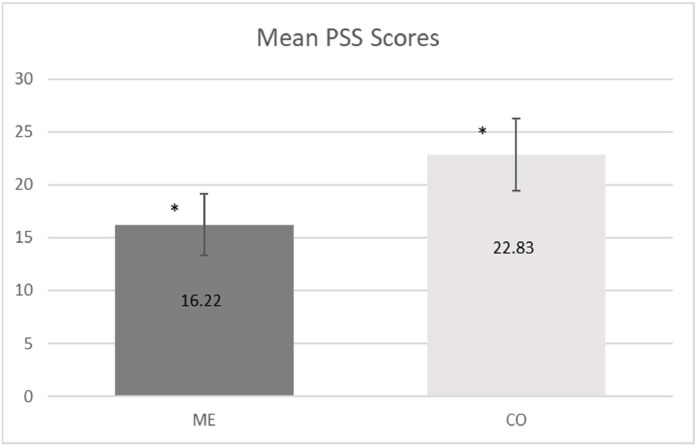
Mean Perceived Stress Scale (PSS) Scores in Meditation Experts (ME) and Controls (CO). * *p* < 0.001.

The internal consistency for the 14 items was good (α = 0.87). The ME group PSS score was significantly lower (ME 16.2 ± 5.2 vs. CO 22.8 ± 7.1, *p* < 0.001), as shown in Fig. 1.

### Resilience

**Fig. 2:**
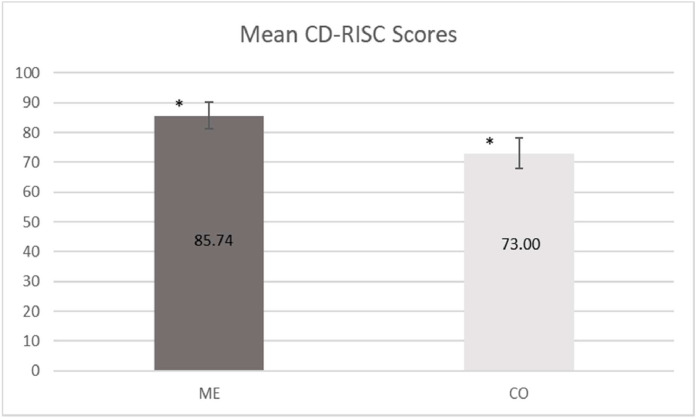
Mean Connor-Davidson Resilience Scale (CD-RISC) Scores in Meditation Experts (ME) and Controls (CO). * *p* < 0.001.

The internal consistency for the 25 items of the questionnaire was excellent (α = 0.91). CD-RISC scores for ME were significantly higher than the CO (ME 85.7 ± 9.2 vs. CO 73.0 ± 9.4, *p* < 0.01), as shown in Fig. 2.

### Mood states

The overall internal consistency for the 16 items of the questionnaire was negative (α = −0.41), violating the suppositions of the reliability model. So it was not considered as a valuable result. The anxiety factor of the VAMS scores was significantly higher, and the mental sedation was lower in the ME group (Table 2). The other VAMS factors, physical sedation, and other feelings and attitudes were similar in the two groups.

**Table 2:** WHOQOL-BREF, VAMS and IRI parametric and non-parametric data results.

	Parametric quantitative data results				Non-parametric quantitative data results			
Scale	Domain	ME	CO	p (Student *t*-test)	Domain	ME	CO	p (MW)
		Mean (SD)	Mean (SD)			Median (Q25‒Q75)	Median (Q25‒Q75)	
WHO QOL BREF	Physical	84.7 (8.7)	78.5 (12.4)	0.048	**Q1**	4.0 (4.0‒5.0)	4.0 (4.0‒4.0)	0.006
	Social	83.2 (8.0)	77.5 (12.3)	0.059				
	Environmental	83.7 (8.5)	75.5 (8.6)	0.002	**Q2**	4.0 (4.0‒4.0)	4.0 (3.0‒4.0)	0.206
	Psycho-logical	83.3 (6.6)	75.2 (9.3)	<0.001				
VAMS	Physical Sedation	32.7 (3.7)	30.7 (4.4)	0.082	**Anxiety**	14.0 (13.1‒14.9)	11.4 (10.4‒12.3)	0.002
	Mental Sedation	3.2 (1.8)	5.3 (3.0)	0.006				
	Other	8.9 (1.6)	9.9 (2.5)	0.217				
IRI	Cognitive	28.1 (3.4)	27.3 (3.0)	0.324	**Emotional**	30.0 (26.5‒34.0)	26.0 (25.0‒29.0)	0.004
	Behavioral	18.0 (4.2)	18.7 (3.4)	0.545	**Sum**	75.0 (71.5‒81.5)	71.0 (70.0‒77.0)	0.065

WHOQOL BREF, World Health Organization Quality of Life BREF Questionnaire; VAMS, Visual Analogue Mood Scale; IRI, Interpersonal Reactivity Index of Davis; ME, Meditation Experts; CO, Controls; SD, Standard Deviation; Q25, Quartile 25; Q75, Quartile 75; MW, Mann Whitney.

### Empathy

The internal consistency for the 21 items of the questionnaire was questionable (α = 0.65). Only in the emotional subscale of the IRI, the ME had higher scores than the CO group. There were no statistically significant differences in IRI Sum scores and in the other IRI subscales scores (cognitive and behavioral) (Table 2).

### Quality of life

The overall internal consistency for the 26 items of the questionnaire was good (α = 0.80). The general quality of life perception (Q1), and scores of WHOQOL-BREF physical, psychological, and environmental domains were significantly higher in the ME group (Table 3). Scores on health satisfaction (Q2) and social domain of WHOQOL-BREF were similar in the two groups (Table 2).

**Table 3:** Individual Lifestyle Profile (PEVI) results.

Parametric quantitative data results				Non-parametric quantitative data results			
Domain	ME	CO	p (Student *t*-test)	Domain	ME	CO	p (MW)
	mean (SD)	mean (SD)			median (Q25‒Q75)	median (Q25‒Q75)	
Stress management	2.9 (0.7)	2.0 (0.8)	<0.001	Nutrition	3.0 (2.3‒2.5)	2.0 (1.3‒2.7)	<0.001
				Social relationships	3.0 (2.5‒3.8)	2.7 (2.0‒3.0)	0.024
Physical activity	2.5 (0.8)	1.6 (0.7)	<0.001	Preventive behavior	2.7 (2.0‒3.7)	3.0 (2.0‒3.0)	0.563
				General behavior	43.0 (33.5‒51.0)	31.0 (28.0‒38.0)	<0.002

ME, Meditation Experts; CO, Controls; SD, Standard Deviation; Q25, Quartile 25; Q75, Quartile 75; MW, Mann Whitney.

### Lifestyle profile

The internal consistency for the 15 items of the questionnaire was good (α = 0.89). The results of PEVI are shown in Table 3. The ME group had a significantly better general behavior score, and also higher scores on all lifestyle domains except for preventive behavior.

## Discussion

To the best of our knowledge, this is the first study to evaluate the psychological characteristics, quality of life, and lifestyle profile of experienced practitioners on Gurusakásha.

The meditators group had lower levels of perceived stress and scored better on resilience, and stress management. This suggests that Meditation on the Essence of Self promotes the relaxation response with an indirect positive effect on health and well-being. The present results are consistent with a previous study, which has demonstrated that a six-week tantric yoga program modulates cortisol response and reduces perceived stress.^20^

The ME group had better scores on the PEVI scale, and also on its stress management, nutrition, physical activity, and social relationships subscales. The authors hypothesized that it may be related to the ancient tantric tradition, which comprises not only meditation but also the practice of Yoga asanas and a specific dietary pattern (e.g., lacto-vegetarianism and avoidance of alcohol). The ME healthier lifestyle profile may have influenced their better results on stress levels, resilience, and quality of life.

The ME showed better quality of life not only in the majority of the WHOQOL-BREF domains but also in the quality-of-life perception. The absence of statistical difference between groups in the social domain may be related to the sample size since a significant difference was achieved in the PEVI social domain. This finding is also in accordance with the findings of other types of meditation.^1^

The VAMS results were not considered since the internal consistency of the test responses was negative. Cronbach’s alpha reliability coefficient will be negative whenever there is greater within-subject variability than between-subject variability.^21^ This reflects bad measuring as far as internal consistency is concerned, and VAMS scores should be considered unreliable.^21^ However, VAMS is an established instrument, and it has been used in many other researches on meditation. The authors suggest that the lack of reliability is probably due to participants' factors like bad interpretation and misunderstandings. This can explain why the ME group scored higher in the anxiety factor of the VAMS, but not in the other VAMS factors. Another possible hypothesis for this result is that the ME group, to participate in this research, had to travel from their homes in distant cities in Brazil to another city where their brain activity was evaluated in the fMRI (data not shown). All the controls were from the same city where the research was conducted. Therefore, the long journey between cities, the absence of a place to stay overnight, and the long wait for the fMRI scan may have influenced the result of the VAMS questionnaire.

Most IRI subscale scores, except the emotional subscale, had no statistically significant differences between the ME and CO groups. The authors found the internal consistency of the IRI questionnaire to have questionable reliability (Cronbach's α = 0.65). The authors then investigated possible explanations and found that there are differences in the cultural tradition and philosophical background of Gurusakásha meditators, who have a different understanding than Western researchers about emotions. The authors of the IRI scale interpret certain emotional reactions as empathetic, such as in “When I see someone who badly needs help in an emergency, I go to pieces”. But ancient tantra tradition interprets “going to pieces” as an inappropriate action in an emergency. An appropriate action for an expert meditator is to be in control of the emotions, expressing at the same time compassion and courage to help someone in an emergency. Despite the fact that IRI had been utilized in many meditation practitioners' studies, and thus was chosen in this research, it did not capture the emotional reactions of tantric meditation practitioners. This was one of the main learning of this study, and the authors recommend authors in future research to check each question of the psychological inventories with a senior meditation instructor in order to avoid misunderstandings, especially when studying practitioners of third-phase meditation.^10^

The main limitation of this study is the relatively small sample size. When taking in account experienced meditators on the Essence of Self, and the majority of the third phase meditations,^10^ scarcity of such experienced practitioners is common. Thus, the size of the sample should not invalidate the need to investigate the meditation technique, but conclusions on efficacy and effects should be interpreted cautiously.

The present study is limited to only suggesting a relation between higher resilience and lower perceived stress to the meditation practice of *Gurusakásha.* It is not possible to conclude any effect of this meditation since more resilient and less stressed people could look for meditation practices more often than other psychological profiles.

Recent research has made efforts through experimental evidence to incorporate the benefits of ancestral Eastern culture and practices in scientific knowledge.^22^ Our paper reinforces this rapprochement between East and West, with quantitative basis, so that the benefits of these ancient practices are not lost.

## Conclusions

The present results showed that this group of experienced meditators on the Essence of Self have more ability to manage stress, higher levels of resilience, and better quality of life and lifestyle profile compared to non-mediators. Some components of their lifestyle may be associated with these characteristics, such as being more physically active and having a healthier diet.

Further research should be done to investigate the clinical effects and behavior change of the Meditation on the Self technique on healthy and diseased individuals. Nonetheless, considering psychological stress, emotional dysregulation, and unhealthy lifestyle behaviors are at the root of the global burden of noncommunicable diseases which are now the leading cause of disability-adjusted life years and account for almost 2/3 of all deaths.^23^, ^24^, ^25^ Gurusakásha is a promising intervention to investigate.

## Funding

Aproved to HC LIMs-FMUSP nov-17 Grant (#83498).

## CRediT authorship contribution statement

**Paula Ricci Arantes:** Conceptualization, Investigation, Resources, Data curation, Formal analysis, Writing – original draft, Writing – review & editing, Project administration. **Tatiana Caccese Perrotti:** Formal analysis, Data curation, Software, Writing – original draft, Writing – review & editing. **Susan Andrews:** Conceptualization, Investigation, Resources, Writing – review & editing, Supervision. **Ernesto Sasaki Imakuma:** Conceptualization, Writing – original draft, Writing – review & editing. **Rosana Aparecida de Oliveira Maurelli:** Investigation, Data curation. **Emmanuel A. Burdmann:** Conceptualization, Writing – review & editing, Supervision.

## Declaration of competing interest

The authors declare no conflicts of interest.
